# Digital transformation of everyday lives of older Swiss adults: use of and attitudes toward current and future digital services

**DOI:** 10.1007/s10433-021-00677-9

**Published:** 2022-01-11

**Authors:** Alexander Seifert, Neil Charness

**Affiliations:** 1 Center of Gerontology, University of Zurich, Zurich, Switzerland; 2 School of Social Work, Institute for Integration and Participation, University of Applied Sciences and Arts Northwestern Switzerland, Riggenbachstrasse 16, 4600 Olten, Switzerland; 3 Florida State University, Tallahassee, USA

**Keywords:** ICT, Digital services, Technology acceptance, Older adults

## Abstract

Digital (consumer) services, such as ticket machines, self-checkout, and online reservations, have become increasingly important in modern society. Studies on adoption of these services and openness to using future public digital services (e.g., online voting, online taxes, electronic patient records) have mostly focused on younger adults or nonrepresentative samples among older adults. Therefore, two important questions remain that can best be addressed with representative sampling: To what extent do older adults use or are willing to use current and future digital services in their everyday lives? How do older adults evaluate the ease of use of these services?. The study included data on use of current and future digital services among a large Swiss sample of 1149 people age 65 years and older (mean age: 74.1 years, SD: 6.69). Descriptive and multivariate analyses showed that (a) established services such as cash machines were used more often than new services, such as self-checkout apps or machines. (b) Perceived ease of use is related to age, socioeconomic status, health, and interest in technology. (c) Only 8.9% had an overall positive attitude toward these digital services, and this attitude was predicted by age, gender, socioeconomic status, and interest in technology. (d) Participants were more often open to filing taxes online than voting online, and openness was predicted by age, income, and interest in technology. Today, mainly older adults with a high interest in technology use digital services. Nevertheless, potential for greater use is evident.

## Introduction

The acceptance and use of modern information and communication technologies (ICTs), such as the internet and digital services in everyday life contexts (e.g., cash machines, ticket machines, or self-checkout apps), have become key features in everyday life. These technologies can be helpful, especially for older adults (age ≥ 65 years) by helping them maintain social functioning and mental health, ensure their independence, and engage with important life goals (Schulz et al. 2015; Cotten 2021). Although ICT use levels are high in Europe and most other developed countries, a digital divide continues to exist between younger (age < 65 years) and older age groups (age ≥ 65 years; Hunsaker and Hargittai 2018; Anderson et al. 2019). Nevertheless, these digital services have the potential to be useful tools for performing everyday life tasks in a digital-oriented everyday context, which is more often discussed today as a way to promote healthy lifestyles among older adults (World Health Organization [WHO] 2017).

Digital consumer services (short: digital services) are any services “normally provided for remuneration, at a distance, by electronic means and at the individual request of recipient of services” (European Parliament 2015, p. 1). Everyday life digital services include ticket machines, self-checkout services, cash machines, and online reservations. An adult who uses these services is called a “digital consumer” (Dey et al. 2020), a new role for consumers in a digital-oriented everyday life world. However, little is known about the current use and evaluation of the ease of use of digital (consumer) services, attitudes toward these digital services, and openness to using future digital public services among the general population of people age 65 years and older. The present study attempts to close this gap in knowledge by presenting representative data from Switzerland.

## Use of digital services among older adults

We live in a society where daily life includes technological innovations and digital content, such as reading breaking news about the coronavirus on a tablet, recording daily activity behavior that can be read from a smartwatch pedometer, or paying digitally with a smartphone. This digital transformation of everyday life experiences yields opportunities to maintain daily life situations; however, participating in this digital society can also be problematic (Lupton 2015), as it is necessary to adapt to all technical innovations and have the skills to be digitally savvy. The degree of digitalization of ICTs and its consequences, therefore, are challenging, because we have to keep in mind that the “digital surroundings” (Dufva and Dufva 2019) are not familiar to everyone in society. For instance, specific portions of the US population (as well as worldwide) do not have direct or immediate access to such new technologies and thus have fewer opportunities to attain additional benefits in their daily lives (Francis et al. 2019). This “digital divide” can be understood as a globally applicable term for the “perceived gap between those who have access to the latest information technologies and those who do not” (Compaine 2001, p. xi). In addition to sociodemographic characteristics (e.g., age, gender, education, income) and individual factors (e.g., health, attitudes toward technologies, anxiety related to using ICTs), environmental factors, such as the ICT infrastructure and the wealth status of the region, shape this gap (Warschauer 2004; Korupp and Szydlik 2005; Wang et al. 2011; Mitzner et al. 2019).

Compared to younger adults, who are more often very familiar with the newest technologies, older adults, in general, are less familiar and skilled in using ICTs. About three-fourths of individuals age 65 years and older in the USA report using the internet compared to 100% for those age 18–29 years, and older individuals also are less likely to have access to broadband than are younger age groups, 55% compared to 77% for those age 18–29 years (Pew Research Center 2019). Thus, too many older adults end up being left behind in the digitalization of society (Seifert et al. 2018).

Regarding use of digital services, such as online reservations or self-checkout machines, researchers have emphasized the “digital divide” between younger and older adults, and the need for representative data regarding use of and attitudes toward digital services among older adults (Nunan and Di Domenico 2019). As Nunan and Di Domenico (2019) argue, less is known about use of everyday life digital services among older adults, which leads to three questions: (a) What is the use and readiness to use of digital services (e.g., cash machines, public transportation ticket machines, contactless payments, self-scanner cash registers in grocery stores, and self-checkout apps or machines) among the population age 65 years and older? (b) What is the perceived usability of current digital services? (c) What attitudes toward current and future digital services do older adults have?

## Research objectives

Given this background, in the present research, we were first interested in evaluating actual use and perceived ease of use of everyday life digital services (i.e., public transportation ticket machines, cash machines, self-scanner cash registers in grocery stores, contactless payment, and self-checkout apps or machines) among older adults age 65 years and older. Based on previous work (Berkowsky et al. 2017; Mitzner et al. 2019; Pirhonen et al. 2020), the likelihood of using digital services such as cash machines and online services is high for socially active, younger, educated, and relatively wealthy older adults than their age peers with lower socioeconomic status. We assumed that age, education, income, subjective health, and interest in technology would predict use and perceived ease of use.

Our second interest in this study was older adults’ attitudes: What attitudes toward digital services are found in the older population? We were also interested in the predictors of these attitudes. A study from Denmark (Siren and Knudsen 2017) found in the background of the well-established technology acceptance model (Venkatesh and Davis 2000) and unified theory of acceptance and use of technology (Venkatesh et al. 2003) that nonuse of ICT often results from a lack of interest, knowledge, and willingness to use it rather than from material or cognitive deficiencies, showing that use is predicted by attitudes toward this use. Therefore, it is appropriate to consider factors that predict this attitude toward digital services. In the background of the technology acceptance model, Chen and Chan (2014) developed the senior technology acceptance model (STAM) to predict use among older adults. Based on these models, we assumed that age, education, income, subjective health, and interest in technology predict attitudes regarding digital services.

Our third research question was related to use of future digital services: Is there openness to using future digital public services (e.g., filing taxes online, voting online, or ordering passports online) among older adults? We were also interested in the explanation of the differences in older adults’ levels of openness. Based on previous studies and reviews (Powell et al. 2012; Gibson et al. 2016)—showing that, for example, e-voting is not widely offered in European countries, we hypothesized when we collected data shortly before the current COVID-19 pandemic that we would find a low percentage of participants who are open to using digital public services, due to the low visibility and experiences of and with these services. Regarding the expected differences in this openness among the older population, the factors presented in the second research question were considered.

## Design and methods

### Data

For this study, a representative survey of older adults age 65 years and older in Switzerland (Seifert et al. 2020) that was made available for secondary analysis was used. This survey originally investigated use and nonuse of the internet by older adults in Switzerland but included information about digital services. In August and September 2019, 1149 people age 65 years and older in all the language regions (Italian: *n* = 109; French: *n* = 261; German: *n* = 779) of Switzerland were interviewed. Computer-assisted telephone interviews (CATI, *n* = 717) were used, supplemented by a paper-and-pencil survey for households without a telephone connection (*n* = 432). A standardized questionnaire with multiple-choice questions was used. A random sample of the permanent resident population of Switzerland aged ≥ 65 was selected using the AZ-Direct database (address pool based on phone book entries). There were no restrictions on upper age, current internet use, nationality, or type of housing. The study included older adults living in private households across all age groups 65 years and older and had only a small underrepresentation of those 90 years and older. All the participants provided verbal informed consent, and the study was conducted according to the guidelines of the Ethics Committee of University of Zurich (Seifert et al. 2020). Table 1 provides the characteristics of the sample. The respondents ranged in age from 65 to 101 years, with a mean age of 74.1 years (SD = 6.69). Of the sample, 80.2% (*n* = 922) used the internet, and 19.8% (*n* = 227) did not use the internet.

**Table 1:** Description of the sample

Parameter	Scale	Sample (*N* = 1149)	
		*n*	%
Gender	Female	579	51.0
	Male	556	49.0
	No response	14	
Age groups	65–69	344	30.4
	70–74	318	28.1
	75–79	227	20.1
	80–84	145	12.8
	85 +	96	8.5
	No response	19	
Education	Compulsory education	143	13.0
	Secondary	590	53.4
	Tertiary	371	33.6
	No response	45	
Household income (in CHF)	Less than 2001	28	2.9
	2001–4000	254	26.7
	4001–8000	463	48.7
	More than 8000	205	21.6
	No response	199	
Living situation	Lives alone	336	30.9
	Does not live alone	751	69.1
	No response	62	
Living area	Rural area	170	14.8
	Nonrural area	979	85.2
	No response	0	
Internet use	User	922	80.2
	Nonuser	227	19.8

### Measures

Three aspects were used for dependent variables. The first dependent variable was based on information about use and evaluation of ease of use of digital services in everyday life (cash machines, public transportation ticket machines, contactless payment, self-scanner cash registers in grocery stores, and self-checkout apps or machines). Use was measured with the following response options: “always if possible,” “from time to time,” “only if there is no other option,” “never used before but interested,” and “never used before and not interested.” For each digital service, a binary variable was calculated with 1 (1, “always if possible,” to 3, “only if there is no other option”) and 0 (4, “never used before but interested,” and 5 “never used before and not interested”) to differentiate between users and nonusers. Ease of use was measured with a 5-point Likert scale (1 = *very difficult* to 5 = *very easy*) for each service. Additionally, ease of use data was divided into users (ease of use) and nonusers (perceived ease of use).

The second dependent variable was based on attitudes toward digital services, measured with six statements (e.g., “Digital services put the security of my data at risk,” “Digital services save time”; see Table 4). The answers were collected on a 5-point Likert scale (1 = *do not agree at all* to 5 = *fully agree*). A mean scale of all six statements was calculated (*M* = 2.77, SD = 0.834) to have a variable that reflects overall attitudes toward digital services, with higher scores reflecting more positive attitudes toward digital services. Negatively directed statements (“Digital servicers threaten jobs” and “digital services put the security of my data at risk”) were recoded. Cronbach’s alpha for the scale was 0.688 (McDonald’s omega = 0.633).

The third dependent variable was based on information about openness to new or future digital public services. Four public services were presented for evaluation: the ability to (a) … complete and file taxes online, (b) … order identity documents (e.g., passports) via the internet, (c) … have an electronic patient record (for the health care system in Switzerland), and (d) … vote via the internet. The responses were collected with a 5-point Likert scale (1 = *completely unnecessary* to 5 = *absolutely necessary*). A mean scale of all four services was calculated (*M* = 3.05, SD = 1.171) to have a variable that reflects overall openness to new digital public services, with higher scores reflecting more openness toward those services. Cronbach’s alpha for the scale was 0.776 (McDonald’s omega = 0.777).

A set of predictor variables established in previous research was considered to explain ease of use of and attitudes toward digital services and openness to new digital public services. The following sociodemographic variables (see Table 1) were included: gender (1 = female; 0 = male), age (in years), education (1 = primary level; 2 = secondary level; 3 = tertiary level), monthly household income (1 = less than 2000 Swiss Francs [CHF]; 2 = CHF 2000–4000; 3 = CHF 4000–8000; 4 = more than CHF 8000), living situation (1 = live alone; 0 = do not live alone), and residential area (1 = rural area; 0 = nonrural area). To measure satisfaction with personal health (*M* = 3.98, SD = 1.150), a self-report question (“My health is still very good for my age”) was used and measured on a 5-point Likert scale (1 = *does not apply at all* to 5 = *applies fully*). Similar to previous research (Schlomann et al. 2020; Seifert and Schelling 2015), interest in new technology (*M* = 3.16, SD = 1.260) was measured with a self-report question (“I’m very interested in new technology”) on a 5-point Likert scale (1 = *does not apply at all* to 5 = *applies fully*).

### Data analysis

SPSS (version 27) was used for the statistical analyses. In addition to descriptive analyses and *t* test statistics, binary logistic and linear regression analyses were performed to evaluate significant predictors of use of digital services, ease of use, attitudes toward, and openness to use of those services at different levels, including demographics (age, gender, education, income, living situation, and area of residence), subjective health, and interest in new technologies.

## Results

### Use and ease of use of digital services

Regarding the use of digital services in everyday life, 63.9% of the sample stated that they always if possible used a cash machine to get cash, and only 7.4% stated that they had never used one before and were not interested in using it. The results were different for self-scanner cash registers in grocery stores: 15.6% always used it when possible, and 64.4% stated that they did not use it and were not interested in using it in the future (Table 2). Users ranked the services in the following order for “always if possible” (Table 2): (a) cash machines (63.9%), (b) public transportation ticket machines (27.4%), (c) contactless payment (15.7%), (d) self-scanner cash registers (15.6%), and (e) self-checkout apps or machines (3.0%). Ranked by general use (“always if possible” to “only if there is no other option”), public transportation ticket machines were used more often than cash machines; only 9.8% had ever used a self-checkout app (Table 2).

**Table 2:** Use and ease of use of digital services

Devices	Use (%)					Only % of users	Ease of use by users^a^	Perceived ease of use by nonusers^b^
	Always if possible	Occasionally	Only if there is no other option	Never used before but interested	Never used before and not interested	*%*	*M* (SD)	*M* (SD)
Cash machine (bank)	63.9	17.3	7.0	4.5	7.4	88.1	4.53*** (0.824)	3.15*** (1.337)
Public transportation ticket machine	27.4	21.0	22.8	12.6	16.1	71.3	3.50*** (1.132)	2.90*** (1.232)
Contactless payment (e.g., Apple Pay or contactless bank card)	15.7	15.6	7.0	14.7	46.9	38.3	4.32*** (0.981)	3.42*** (1.462)
Self-scanner cash registers in grocery stores	15.6	13.8	9.0	13.8	47.7	38.4	4.21*** (1.061)	3.20*** (1.296)
Self-checkout apps or machines (e.g., books, bikes, e-scooters)	3.0	5.4	1.4	25.8	64.4	9.8	4.00*** (1.021)	3.10*** (1.296)

Devices sorted by frequency of “always if possible.”

*T* test statistics: *p < 0.05. **p < 0.01. ***p < 0.001

^a^Ease of use of users (1, “always if possible,” to 3, “only if there is no other option”): Scale (1 = *very difficult* to 5 = *very easy*)

^b^Perceived ease of use of nonusers (4, “never used before but interested,” and 5, “never used before and not interested”): Scale (1 = *very difficult* to 5 = *very easy*)

When users of these digital services were asked how they evaluated the ease of use of the services, users ranked the services in the following order: (a) cash machines, (b) contactless payment, (c) self-scanner cash registers, (d) self-checkout apps or machines, and (e) public transportation ticket machines. Among nonusers of these digital services, the services were ranked as follows: (a) contactless payment, (b) self-scanner cash registers, (c) cash machines, (d) self-checkout apps or machines, and (e) public transportation ticket machines. Nevertheless, over all the services, users (people who already used the services) reported higher ease of use than nonusers did (they perceived the ease of use lower) in general (Table 2).

In addition to an examination of use of these digital services, the predictors of this evaluation were assessed. Binary logistic regression models (Table 3) were created to predict the factors for use of the five different services. The independent variables included nontechnical variables (age, gender, education, income, living situation, subjective health) and interest in technology. The first model (public transportation ticket machine) showed that age and education were statistically significant predictors. Younger participants and participants with higher education levels are more often among the group of users. The second model (cash machine) showed that age, education, and interest in technology were statistically significant predictors. Younger participants, participants with higher education levels, and people with higher levels of interest in technology are more often among the group of users. The third model (self-scanner cash registers) showed that age, education, income, and interest in technology were statistically significant predictors. Younger participants, people with higher education levels, people with higher income, and people with higher levels of interest in technology are more often among the group of users. The fourth model (contactless payment) showed that age, education, income, and interest in technology were statistically significant predictors. Younger participants, people with higher education levels, people with higher income, and people with higher levels of interest in technology are more often among the group of users. The last model (self-checkout apps or machines) showed that age, education, and rural area were statistically significant predictors. Younger participants, people with higher education levels, and people who did not live in rural areas were more often among the group of users.

**Table 3:** Prediction of use, ease of use, and perceived ease of use based on binary logistic regression and linear regression analysis

Predictors	Public transportation ticket machine			Cash machine (bank)			Self-scanner cash registers in grocery stores		
	Use^a^	Ease of use^b^	Perceived ease of use^c^	Use^a^	Ease of use^b^	Perceived ease of use^c^	Use^a^	Ease of use^b^	Perceived ease of use^c^
	OR	Beta	Beta	OR	Beta	Beta	OR	Beta	Beta
Age	0.955***	0.038	− 0.234**	0.919***	− 0.011	− 0.041	0.926***	− 0.030	− 0.115*
Female (ref. male)	0.712	0.021	0.001	0.959	0.046	0.043	0.785	0.044	0.008
Education^d^	1.465**	0.112**	0.097	2.266***	0.058	0.234	1.570***	0.035	0.090
Income^e^	1.154	0.161***	0.076	1.175	0.128**	0.038	1.430**	0.057	0.050
Lives alone (ref. does not live alone)	1.040	0.124**	0.079	0.871	0.079	− 0.121	0.707	0.078	0.016
Rural area (ref. nonrural area)	0.831	− 0.011	− 0.111	0.912	− 0.001	0.008	0.891	− 0.013	− 0.052
Subjective health^f^	1.081	0.101*	0.041	1.004	0.103**	− 0.066	1.042	0.091	0.094
Interest in technology^g^	1.046	0.088*	0.204**	1.348**	0.054	0.013	1.262***	0.028	0.101
Model fit	CS (8, 886) = 41.280; *p* < 0.001; NR^*2*^ = 0.067	*F*(8, 644) = 6.582; *p* < 0.001; corrected *R*^2^ = 0.065	*F*(8, 183) = 3.779; *p* < 0.001; corrected *R*^2^ = 0.108	CS (8, 901) = 80.242; *p* < 0*.*001; NR^2^ = 0*.*178	*F*(8, 803) = 4.149; *p* < 0.001; corrected *R*^2^ = 0.030	*F*(8, 67) = .653; *p* = 0.730; corrected R^2^ = 0.004	CS (8, 895) = 112.772; *p* < 0*.*001; NR^2^ = 0.159	*F*(8, 352) = 0.875; *p* = 0.538; corrected *R*^2^ = 0.003	*F*(8, 320) = 2.642; *p* = 0.008; corrected *R*^2^ = 0.039

Predictors	Contactless payment			Self-checkout apps or machines		
	Use^a^	Ease of use^b^	Perceived ease of use^c^	Use^a^	Ease of use^b^	Perceived ease of use^c^
	OR	Beta	Beta	OR	Beta	Beta
Age	0.952***	0.016	− 0.181**	0.945**	0.125	− 0.172**
Female (ref. male)	1.116	0.146*	− 0.083	0.681	− 0.076	− 0.021
Education^d^	1.520***	0.104	0.033	1.575*	0.005	0.103
Income^e^	1.422**	0.094	0.114	1.274	0.088	0.092
Lives alone (ref. does not live alone)	0.880	− 0.018	0.080	0.806	− 0.060	0.079
Rural area (ref. nonrural area)	0.860	− 0.110*	− 0.072	0.465**	− 0.158	− 0.070
Subjective health^f^	0.949	0.078	0.175**	1.016	− 0.052	0.070
Interest in technology^g^	1.179*	0.103	0.131*	1.219	0.217	0.119*
Model fit	CS (8, 887) = *82.357; p* < 0*.*001; NR^2^ = 0.120	*F*(8, 335) = 2.990; *p* = 0.003; corrected *R*^2^ = 0.045	*F*(8, 283) = 5.457; *p* < 0.001; corrected *R*^2^ = 0.112	CS (8, 875) = 39.400; *p* < 0*.*001; NR^2^ = 0.089	*F*(8, 84) = 1.245; *p* = 0.285; corrected *R*^2^ = 0.023	*F*(8, 321) = 4.351; *p* < 0.001; corrected *R*^2^ = 0.077

^*^p < 0.05. **p < 0.01. ***p < 0.001

^a^Use (scale: 1 = *users*, 0 = nonusers)

^b^Ease of use (only users, scale: 1 = *very difficult* to 5 = *very easy*)

^c^Perceived ease of use (only nonusers, scale: 1 = *very difficult* to 5 = *very easy*)

^d^Education (3 = tertiary, 2 = secondary, 1 = primary level)

^e^Household income (1 = less than CHF 2000; 2 = 2000–4000, 3 = 4001–8000, 4 = more than CHF 8000)

^f^Subjective health (1 = *does not apply at all* to 5 = *fully applies*)

^g^General interest in technology (1 = *does not apply at all* to 5 = *fully applies*)

In addition to a descriptive examination of ease of use and perceived ease of use, the predictors of this evaluation were assessed with linear regression models with the same independent variables used for the predictors of use models (Table 3). The ease of use model of users of public transportation ticket machines revealed that income, education, living alone, subjective health, and interest in technology were statistically significant predictors, whereas the other independent variables were not predictors in the multivariate model. In contrast, the perceived-ease-of-use model for nonusers of public transportation ticket machines revealed that age and interest in technology were statistically significant predictors. The predictors of ease of use of cash machines among users were income and subjective health, whereas the model for perceived ease of use was not statistically significant. The only predictor of perceived ease of use of self-scanner cash registers among nonusers was age (people who were younger perceived the use as easier); the model for ease of use for this digital service was not statistically significant. The predictors of ease of use of contactless payment among users were gender and rural areas (women and people living in nonrural areas evaluated the service as easier to use than men and people living in rural areas), whereas the predictors for perceived ease of use among nonusers were age, subjective health, and interest in technology (younger participants, people with better subjective health, and people with higher levels of interest in technology perceived the service as easier to use). Predictors of perceived ease of use of self-checkout apps or machines among nonusers were age and interest in technology (people who were younger and people who were interested in technology perceived the use as easier); the model for ease of use for this digital service was not statistically significant.

### Attitudes toward digital services

To evaluate attitudes toward digital services, six statements about digital services were included in the survey (Table 4). The two negative statements (“Digital services threaten jobs” and “digital services put the security of my data at risk”) were more often evaluated with higher values; however, the other statements were often evaluated positively (with mean values higher than the mean of the 1–5 scale). Male participants and younger participants (age 65–79 years) had statistically significantly higher positive attitudes than female participants and older participants (age 80 years and older; Table 4). Furthermore, people from the German-speaking part of Switzerland (*M* = 2.86) had a more positive attitude in general (total scale) than the people from the French- (*M* = 2.58) or Italian-speaking (*M* = 2.69) part. When the sample was separated into three attitude groups, we found that 8.9% had positive attitudes (*M* = 4.0–5.0), 69.9% had ambivalent attitudes (*M* = 2.1–3.9), and 21.2% had negative attitudes (*M* = 1.0–2.0).

**Table 4:** Attitudes toward digital services

Statements	Scale^a^	Factor	Female	Male	65–79 years	80 + years	German-Swiss	French-Swiss	Italian-Swiss
	*M* (SD)	Factor loading	*M*	*M*	*M*	*M*	*M*	*M*	*M*
1. Digital services threaten jobs	3.830 (1.321)	0.835	4.06***	3.60***	3.82	3.89	3.67***	4.13***	4.27***
2. Digital services put the security of my data at risk	3.710 (1.223)	0.839	3.90***	3.54***	3.75	3.62	3.60***	3.95***	4.02***
3. Digital services save time	3.510 (1.332)	0.837	3.38**	3.65**	3.58**	3.24**	3.54	3.38	3.63
4. Digital services are more accessible than their alternatives	3.180 (1.272)	0.847	3.02***	3.33***	3.26***	2.79***	3.23	2.99	3.22
5. Digital services are easier to manage than their (offline) alternatives	2.800 (1.302)	0.803	2.61***	3.00***	2.88***	2.49***	2.82	2.78	2.72
6. Digital services are cheaper for me (save money)	2.750 (1.423)	0.735	2.42***	3.04***	2.84***	2.34***	2.81**	2.43**	2.92**
Total scale^b^	2.775 (0.834)	Cronbach alpha = 0.688 McDonald’s omega = 0.633	2.57***	2.98***	2.83***	2.54***	2.86***	2.58***	2.69***

Statements sorted by mean

*T* test statistics/One-Way-ANOVA: *p < 0.05. **p < 0.01. ***p < 0.001

^a^Scale (1 = *do not agree at all* to 5 = *fully agree*)

^b^For “total scale” statements, (1) and (2) were reversed

In addition, the multivariate predictors of this evaluation were assessed. Hierarchal linear regression models (Table 5) were created to differentiate between nontechnical variables (age, gender, education, income, living situation, health) and interest in technology. The first model (1A) showed that gender, income, age, and education were statistically significant predictors, whereas the other independent variables were not predictors in the full model. Younger participants, male participants, participants with higher education levels, and participants with higher income reported higher positive attitudes toward digital services. The full model (1B) revealed that in addition to gender, age, education, and income, interest in technology was a statistically significant predictor. People who reported being interested in technology were more likely to have higher positive attitude values.

**Table 5:** Prediction of attitudes toward digital services and openness to using digital public services based on linear regression analysis

Predictors	Attitudes toward digital services^a^		Openness to using digital public services^b^		
	Model 1A	Model 1B	Model 2A	Model 2B	Model 2C
	Beta	Beta	Beta	Beta	Beta
Age	− 0.101**	− 0.080*	− 0.170***	− 0.154**	− 0.118**
Female (ref. male)	− 0.198***	− 0.142***	− 0.078*	− 0.024	0.032
Education^c^	0.085*	0.070*	0.060	0.033	0.014
Income^d^	0.136***	0.093*	0.147***	0.112**	0.069
Lives alone (ref. does not live alone)	0.020	0.006	0.001	− 0.011	− 0.007
Rural area (ref. nonrural area)	− 0.008	− 0.014	− 0.029	− 0.034	− 0.025
Subjective health^e^	0.019	0.021	0.006	0.013	− 0.007
Interest in technology^f^		0.251***		0.235***	0.139***
Attitudes toward digital services^a^					0.391***
Model fit	*F*(7, 859) = 15.031; *p* < 0.001; corrected *R*^2^ = 0.103	*F*(8, 852) = 20.769; *p* < 0.001; corrected *R*^2^ = 0.157	*F*(7, 894) = 12.257; *p* < 0.001; corrected *R*^2^ = 0.081	*F*(8, 890) = 17.338; *p* < 0.001; corrected *R*^2^ = 0.128	*F*(9, 847) = 32.470; *p* < 0.001; corrected *R*^2^ = 0.251

^*^p < 0.05. **p < 0.01. ***p < 0.001

^a^Attitudes toward digital services (Total scale of attitudes; see Table 4, scale: 1 = *do not agree at all* to 5 = *fully agree*)

^b^Openness to using future digital public services (total scale of openness; see Table 6, scale: 1 = *completely unnecessary* to 5 = *absolutely necessary*)

^c^Education (3 = tertiary, 2 = secondary, 1 = primary level)

^d^Household income (1 = less than CHF 2000; 2 = 2000–4000, 3 = 4001–8000, 4 = more than CHF 8000)

^e^Subjective health (1 = *does not apply at all* to 5 = *fully applies*)

^f^General interest in technology (1 = *does not apply at all* to 5 = *fully applies*)

### Openness to using public services

Openness to using new digital public services was more favorable for online taxes than for online voting, resulting in the following: (a) a mean of 3.39 for doing taxes online, (b) a mean of 3.15 for ordering identity documents online, (c) a mean of 2.97 for having an electronic patient record, and (d) a mean of 2.76 for voting online (Table 6). Male participants and younger participants (age 65–79 years) had statistically significant higher levels of openness to use than female participants and older participants (age 80 years and older; Table 6). Regarding “online voting,” we found a statistically significant correlation (Pearson *r* = –0.179, *p* < 0.001) with the statement “digital services put the security of my data at risk,” which shows people who have the feeling that digital services are not secure were less open to using online voting. Furthermore, a statistically significant correlation (Pearson *r* = 0.464, *p* < 0.001) with the general attitudes toward digital services scale was found, showing that people with a positive attitude toward digital services, in general, are more open to using digital public services.

**Table 6:** Openness to using future digital public services

Digital services (The ability to …)	Scale^a^	Female	Male	65–79 years	80 + years
	*M *(SD)	*M*	*M*	*M*	*M*
… Complete and file taxes online	3.390 (1.497)	3.20***	3.57***	3.54***	2.77***
… Order identity documents (e.g., identity card or passport) via the internet	3.150 (1.532)	2.91***	3.37***	3.24***	2.73***
… Have an electronic patient record	2.970 (1.474)	2.78***	3.17***	3.04**	2.72**
… Vote via the internet	2.760 (1.548)	2.62**	2.90**	2.82*	2.54*

Services sorted by mean

*T* test statistics: *p < 0.05. **p < 0.01. *** *p* < 0.001

^a^Scale (1 = *completely unnecessary*, 5 = *absolutely necessary*)

In addition to descriptively examining openness to using these digital services, the multivariate predictors of this evaluation were assessed. Hierarchal linear regression models (Table 5) were created to differentiate between nontechnical variables and technical independent variables. The first model (2A) showed that age, gender, and income were statistically significant predictors. Younger participants, male participants, and participants with higher income were more open to using these digital public services than older participants, female participants, and participants with lower income. The second model (2B) revealed that in addition to age and income, interest in technology was a statistically significant predictor, whereas the other independent variables were not predictors in the multivariate model. The full model (2C) revealed that in addition to age and interest in technology, attitudes toward digital services was a statistically significant predictor, whereas the other independent variables were not predictors in the multivariate model. People who reported being interested in technology, younger participants, and participants with higher positive attitudes toward digital services were more likely to be open to using new public digital services.

## Discussion

The findings are based on a sample of 1149 people age 65 years and older living in Switzerland. The aim of the study was to add to a relevant and growing research field that addresses everyday use of digital services by older adults as well as their openness to using future digital public services. A major but not unexpected finding for the sample was that older adults showed a noteworthy level of digital services use in Switzerland (compared to data about internet use in our sample 80% use the internet, whereas 53% in Europe aged 50 years and older do (König and Seifert 2020)); however, the same older adults showed some problems with the use of these digital services. In addition, the study showed that among the participants a considerable number of people have positive attitudes toward existing digital services, but only a small number of participants are open to using future digital public services.

The first research question addressed use and evaluation of ease of use of digital services (cash machines, public transportation ticket machines, contactless payment, self-scanner cash registers in grocery stores, and self-checkout apps or machines), technologies that especially during the current COVID-19 pandemic are important, because in-person services (such as bank counters or cash registers) have increasingly been converted to digital services (such as cash machines or self-scanner cash registers in grocery stores). The study showed that established services such as cash machines and public transportation ticket machines are often used in everyday life, whereas shortly before the first COVID-19 cases and deaths were reported in Switzerland, digital services such as self-scanner cash registers in grocery stores and self-checkout apps or machines were used by only a minority. Nevertheless, users of these digital services evaluated them as easier to use than nonusers of these services perceived the services’ ease of use. When people used the digital services successfully, they evaluated them as easier to use. In particular, public transportation ticket machines were evaluated as less easy to use than all the other services, reminding us that even established services are not easy to use on one’s own, especially in a sample of older adults. The complexity of the options seems important: Cash machines have mostly one main target or option (cash withdrawal), whereas the machines for purchasing public transportation tickets offer a variety of sophisticated trip options and discount options.

After the set of established predictors was considered, the multivariate analyses revealed different statistically significant predictors for overall use of digital services. For all digital services, age and education, and to some extent, income (self-scanner cash registers, contactless payment) and interest in technology (not for public transportation ticket machines and self-checkout apps or machines) were statistically significant predictors. Younger participants, people with higher socioeconomic status, and people with higher levels of interest in technology are more often among users than among nonusers of digital services; emphasizing age and socioeconomic effects on technology adaption (Korupp and Szydlik 2005; König and Seifert 2020). Compared with previous studies (Chen and Chan 2014), we can show that age, education, and interest in technology are factors for technology adaption; however, contrary to their finding with a Hong Kong sample, we do not find self-reported health characteristics within our study as significant predictors, perhaps because their study included health technology items not assessed here.

The multivariate analyses for ease of use among users of digital services showed that different statistically significant predictors existed (education, income, living alone, subjective health, interest in technology, rural area, and gender) depending on the application. Subjective health was a predictor for ease of use of public transportation ticket machines and cash machines in addition to socioeconomic predictors. Nevertheless, age was not a statistically significant predictor for ease of use among all digital services. This result shows that age, among users, is not a predictor of ease of use, but health barriers and potentially other predictors that the present study could not include with the available questionnaire are. For nonusers of digital services, the multivariate analyses showed that age was a statistically significant predictor for all five services (not for cash machines), emphasizing the age effect on perceived ease of use; older participants perceived less ease of use than younger participants. Moreover, interest in technology was a statistically significant predictor for perceived ease of use for public transportation ticket machines, contactless payment, and self-checkout apps or machines. People with higher levels of interest in technology perceived ease of use as higher than people with low levels of interest in technology, showing a motivational aspect of investing time in learning new technologies (Kamin et al. 2017; Francis et al. 2019). The importance of interest in technology in the ease-of-use evaluation among older adults has also been found in other studies (Mitzner et al. 2019).

The second research question addressed attitudes toward digital services. The data showing that the participants mostly had ambivalent attitudes. Specifically, 8.9% had positive, 69.9% had ambivalent, and 21.2% had negative attitudes. Therefore, digital services are not always positively evaluated; however, this ambivalence in attitudes toward technologies was also found, for example, regarding the internet among older adults (Seifert and Schelling 2018). We found also a difference between the three language parts of Switzerland, showing that some cultural differences regarding the attitudes toward digital services exist. The multivariate model of attitudes predictors revealed that being younger, being male, having high education and income levels, and having an interest in technology were predictors. This result shows that in addition to sociodemographic factors interest in technology is important to include in future studies. These results regarding interest in new technologies are in line with previous findings (Seifert and Schelling 2018). Nevertheless, the present result also shows that younger people, men, and people with high socioeconomic status see more advantages in digital services.

The last research question addressed openness to using future digital public services. The study revealed that some of the older participants were open to using these services; however, the levels of openness differed according to the application. Participants were, for example, more open to filing taxes online than voting via the internet. This result shows that not all digital public services were evaluated the same. The full multivariate model of this openness revealed that age, interest in technology, and attitudes toward digital services predicted openness. Younger participants, participants with higher positive attitudes toward digital services, and participants who reported an interest in technology were more likely to use these digital public services in the future. The results underpin the age and motivational aspect of the use of future digital services.

To reduce age-related inequalities in digital services and thus increase participation among older adults, more financial resources should be invested in training, educating, and supporting this population group. General recommendations are available to address technological development issues for older adults (Cotten et al. 2016; Czaja et al. 2019), and these recommendations should be used and adapted for the special case of those services. Crucially, developers and providers of digital services should be motivated to closely examine how different designs and forms of content can be tailored to encourage trust and facilitate use among older users. For instance, developers should address older adults’ interest in digital services. If developers consider the end users, this increases the acceptance, usage, and effectiveness of their tools (Merkel and Kucharski 2019). Therefore, involving older adults in participative research is important before developing digital solutions. After developing a tool, it is also important to invest time in educating older adults about its use. A support hotline, printed manuals, and contact partners can assist older adults when necessary.

## Limitations

The data provided only a cross-sectional view of the examined phenomenon. Because it is quite likely—especially in times of physical distancing during a worldwide pandemic—there will be a further increase in digital services use among older individuals, these findings may not reflect future trends or the specific situation regarding the COVID-19 pandemic. Furthermore, in this study, data on important background factors were unavailable, such as technological knowledge, history of use, breadth of daily use, ability to cope with activities of daily life, and personality. Furthermore, the attitudes toward digital services should be tested also with younger adults to check for age-group differences. Therefore, studies with a wider range of participants, variables and longitudinal designs are necessary to examine this topic in more detail.

## Conclusion

The findings show the breadth and diversity of digital services use among older adults. Although established services such as cash machines are becoming more frequently used among this population, use of newer technologies, such as self-scanner cash registers and self-checkout apps or machines, has remained low, which speaks to modest diffusion of those services. Nevertheless, the potential for new adoption, especially regarding the use of some future digital public services, is supported by the data.
